# Impaired vascular responses to parasympathetic nerve stimulation and muscarinic receptor activation in the submandibular gland in nonobese diabetic mice

**DOI:** 10.1186/ar2609

**Published:** 2009-02-06

**Authors:** Ellen Berggreen, Krister Nyløkken, Nicolas Delaleu, Hamijeta Hajdaragic-Ibricevic, Malin V Jonsson

**Affiliations:** 1 Department of Biomedicine, Jonas Liesvei 91, Bergen 5009, Norway; 2 Broegelmann Research Laboratory, Gade Institute, Haukeland Hospital, Bergen 5021, Norway; 3 Ministry of Health, Amiri Dental Center, PO Box 472, Dasman 15455, Kuwait; 4 Department of Medicine, Section for Rheumatology, Gade Institute, Haukeland Hospital, Bergen 5021, Norway; 5 Section for Pathology, Gade Institute, Haukeland Hospital, Bergen 5021, Norway

## Abstract

**Introduction:**

Decreased vascular responses to salivary gland stimulation are observed in Sjögren's syndrome patients. We investigate whether impaired vascular responses to parasympathetic stimulation and muscarinic receptor activation in salivary glands parallels hyposalivation in an experimental model for Sjögren's syndrome.

**Methods:**

Blood flow responses in the salivary glands were measured by laser Doppler flowmeter. Muscarinic receptor activation was followed by saliva secretion measurements. Nitric oxide synthesis-mediated blood flow responses were studied after administration of a nitric oxide synthase inhibitor. Glandular autonomic nerves and muscarinic 3 receptor distributions were also investigated.

**Results:**

Maximal blood flow responses to parasympathetic stimulation and muscarinic receptor activation were significantly lower in nonobese diabetic (NOD) mice compared with BALB/c mice, coinciding with impaired saliva secretion in nonobese diabetic mice (*P *< 0.005). Nitric oxide synthase inhibitor had less effect on blood flow responses after parasympathetic nerve stimulation in nonobese diabetic mice compared with BALB/c mice (*P *< 0.02). In nonobese diabetic mice, salivary gland parasympathetic nerve fibres were absent in areas of focal infiltrates. Muscarinic 3 receptor might be localized in the blood vessel walls of salivary glands.

**Conclusions:**

Impaired vasodilatation in response to parasympathetic nerve stimulation and muscarinic receptor activation may contribute to hyposalivation observed in nonobese diabetic mice. Reduced nitric oxide signalling after parasympathetic nerve stimulation may contribute in part to the impaired blood flow responses. The possibility of muscarinic 3 receptor in the vasculature supports the notion that muscarinic 3 receptor autoantibodies present in nonobese diabetic mice might impair the fluid transport required for salivation. Parasympathetic nerves were absent in areas of focal infiltrates, whereas a normal distribution was found within glandular epithelium.

**Trial registration:**

The trial registration number for the present study is 79-04/BBB, given by the Norwegian State Commission for Laboratory Animals.

## Introduction

Sjögren's syndrome (SS) is a systemic autoimmune disease mainly affecting the exocrine glands, resulting in severe impairment of saliva and tear production. The histopathological hallmarks of the disease are T-cell-dominated and B-cell-dominated focal infiltrates in the salivary glands. It has been suggested that the decrease in salivary flow follows the occurrence of focal lymphoid infiltration, with a considerable delay in time, and that the sole destruction or replacement of glandular tissue by inflammatory cells is not sufficient to explain the severe impairment in salivary secretion [1]. The unclear interrelationship between glandular inflammation and hyposalivation [2,3] has led to research initiatives investigating mechanisms of glandular dysfunction. Autoantibodies inhibiting receptors for neurotransmitter receptors and defective water transport have been proposed [4]. In the salivary glands, blood flow and salivary secretion are under autonomic nervous control of both parasympathetic and sympathetic nerves [5]. During salivation, fluid is transported from the capillaries through the interstitial space, before being secreted by the glandular epithelium [6].

The nonobese diabetic (NOD) mouse strain exhibits immunological, histopathological and physiological characteristics of SS with focal mononuclear cell infiltration of the exocrine glands from approximately 8 weeks of age [7]. The manifestations of overt SS hallmarked by impaired lacrimal and salivary secretion are thought to develop later in life [1]. In the NOD mice, no augmentation of saliva flow rates has been observed after infusion of neuropeptides combined with muscarinic–cholinergic agonist [8], indicating that the hyposalivation observed in NOD mice may, at least in part, be due to a general loss of neurotransmitter responsiveness in salivary glands. On the other hand, an *in vitro *study on human labial gland cells isolated from patients with primary SS has demonstrated similar response to stimulation with acetylcholine and neuropeptides as healthy controls, indicating functional receptor systems [9]. Whether the loss of responsiveness *in vivo *is located on the vascular side or is related to circulating autoantibodies affecting receptor function is unknown.

Changes in receptor expression such as a downregulation of β-adrenergic receptors and their signal transduction response [10], as well as a downregulation of muscarinic receptors [11] and the presence of autoantibodies against muscarinic 3 receptors (M3Rs), have been described in the NOD mice [12]. In contrast, an upregulation of the M3R has been demonstrated in labial salivary gland tissue from patients with SS [13].

Nitric oxide (NO) signalling is activated through muscarinic receptors in the salivary glands [14,15], and NO synthase activity and expression are reported to be decreased in NOD mice [16] – supporting the hypothesis of an impaired neural regulation in the salivary glands in NOD mice. Impaired neurotransmitter release in salivary glands in the MRL/*lpr *mouse, another murine model of SS, has also been reported [17]. Patients with SS have elevated salivary levels of vasoactive intestinal peptide (VIP) and neuropeptide Y (NPY), which are mainly found in parasympathetic and sympathetic nerves, respectively. This finding indicates increased release of VIP and NPY by salivary glands of SS patients [18].

As both vessels and epithelial cells are equipped with muscarinic and adrenergic receptors, and are innervated by autonomic nerves, we hypothesized that the vascular responses to autonomic stimulation may be reduced in the NOD mice. To test this hypothesis, we measured changes in blood flow in the submandibular gland in the response to parasympathetic stimulation in NOD mice, and investigated the contribution of NO to the observed vasodilatation. Furthermore, we measured blood flow responses after muscarinic activation with a simultaneous effect on salivation, and verified the presence of M3R in the wall of blood vessels in the submandibular gland. As potential changes in the autonomic innervation pattern in NOD mice may be directly related to the observed alterations, immunohistochemical detection of both parasympathetic and sympathetic nerves as well as glandular inflammation were included in this study.

## Materials and methods

### Animals

Female NOD mice and BALB/c mice were purchased from Taconic Bomholtgård, (Ry, Denmark) (*n *= 22 + 22) and from Jackson Laboratories (Bar Harbor, Maine, USA) (*n *= 7 + 7), and were kept under standard animal housing conditions at the animal facility of the Department of Biomedicine, University of Bergen, Norway. The experiments were carried out with the approval of the Norwegian State Commission for Laboratory Animals and were approved by the local ethical committee.

The 16-week-old to 18-week-old BALB/c mice and NOD mice were anaesthetized with Hypnorm-Dormicum 0.5 ml/10 g body weight (BW) (Janssen Pharmaceutical, Beerse, Belgium). Pilocarpine hydrochloride and *N*_ω_-nitro-L-arginine-methyl ester (L-NAME) were purchased from Sigma Chemical Co. (St Louis, MO, USA). Two strains of mice were used in this study since Taconic stopped their breeding of NOD mice during the experimental period.

### Blood flow recordings

The BALB/c mice and NOD mice from Taconic (Group 1, *n *= 10 + 10) and from Jackson Laboratories (Group 2, *n *= 7 + 7) (Table 1) were studied in a supine position, and the body temperature was kept at 37 to 38°C with a servocontrolled heating pad. A femoral artery was catheterized for continuous systemic blood pressure recordings with a Gould pressure transducer and recorder, and a submandibular gland dissected free. In 10 BALB/c mice and 10 NOD mice (Group 1), the submandibular duct comprising the lingual nerve with parasympathetic fibres from chorda tympani was placed on an electrode and stimulated electrically. The submandibular gland was chosen since this gland is encapsulated and its main excretory duct easily isolated, thus allowing parasympathetic nerve stimulation. Electrical stimulation was performed with a Grass stimulator (Quincy, MA, USA), giving square wave pulses of 2 milliseconds at 7 Hz; 8 V for periods of 2 to 5 seconds.

**Table 1:** Distribution of nonobese diabetic (NOD) mice and BALB/c mice used

Animal provider	Blood flow recordings	Salivary flow measurements	Focus score/ratio index	Immunofluorescence/immunohistochemistry	NOD mice + Balb/c mice
Taconic Bomholtgård (Group 1)	10 + 10		4 + 4		20
Jackson Laboratories (Group 2)	7 + 7	7 + 7	7 + 7	7 + 7	14
Taconic Bomholtgård		6 + 5^a^			11
Taconic Bomholtgård			6 + 6	6 + 6	12
Total	34	25	34	26	57

Data presented as number of mice. ^a^One BABL/c mouse was lost during salivary stimulation.

A Periflux Model 4001 Master laser Doppler flowmeter (Perimed KB, Järfälla, Sweden) equipped with a needle probe PF 415:10 (fibre diameter 125 μm, with separation 500 μm) was used to measure changes in glandular blood flow in all animals (Group 1 and Group 2). Zero blood flow was calibrated in a zeroing disc and by use of a motility standard giving an output value of 250 perfusion units (PU). We carried out standard calibration of the instruments and fibre-optic probes according to the manufacturer's specifications. The laser probe was positioned with a micromanipulator above the gland on the anterior middle part of the gland and was rotated to the position that gave the largest resting blood flow signal measured in arbitrary PU. The flowmeter set constant was 0.03 and the lower bandwidth was at 20 kHz and at 20 Hz, respectively. All data were stored and analysed using Perisoft computer software (Perisoft 2.1; Järfälla, Sweden).

After one stimulation period (Group 1), a NO synthesis blocker (L-NAME, 90 mg/kg BW) was diluted in 0.05 ml of 0.9% saline and was infused intravenously over a period of 1 minute. Electrical stimulation was repeated 2 to 3 minutes after the end of infusion.

In Group 2 pilocarpine (0.10 mg/100 g BW dissolved in 0.01 ml of 0.9% saline) was infused over 1 minute and the glandular blood flow changes were recorded simultaneously as saliva was collected from the oral cavity into preweighed tubes for 10 minutes (Table 1). This dose was chosen since it has been demonstrated to give reproducible blood flow responses in rat submandibular glands [19]. Measurements of blood flow and systemic blood pressure were measured continuously before, during and after infusions in all animals. After pilocarpine responses were measured and saliva collected, the submandibular gland used for blood flow measurement was removed and fixed in 10% buffered formalin and the contralateral gland was snap-frozen in liquid nitrogen by isopentane. The blood glucose level was tested with blood samples obtained by tail vein puncture before the start of blood flow measurements.

### Stimulated salivary flow measurement

The saliva secretion capacity in response to muscarinic receptor stimulation was assessed in six Balb/c mice and six NOD mice (Taconic; see Table 1) after being fasted for a minimum of 5 hours with water *ad libitum*. Subsequent to intraperitoneal injection of 0.05 mg/100 g BW pilocarpine (dissolved in 0.01 ml of 0.9% saline), saliva was collected from the oral cavity with 20 μl micropipettes for 10 minutes. To prevent asphyxiation, mice were held upright with the tongue extended by a forceps during the experiment [3]. The results are expressed in microlitres of saliva per minute per gram of BW.

### Assessment of hyperglycaemia

NOD mice and the age-matched BALB/c from Taconic were assessed for hyperglycemia using a Reflotron Plus Glucose test kit (Roche Diagnostics, Quebec, Montreal, Canada) and from Jackson Laboratories (Group 2) using the Keto-diabur test 5000 (Roche, Meylan, France). NOD mice with glucose levels higher than 11 mmol/l were considered hyperglycaemic and were excluded from the study [20]. The glucose levels in experimental animals from Taconic and Jackson Laboratories ranged between 3.4 and 8.5 mmol/l and 2.5 mmol/l and 8.2 mmol/l, respectively.

### Immunofluorescence

Salivary gland sections (6 μm) from Group 2 animals (Table 1) were incubated overnight with polyclonal antibody to M3R (Santa Cruz Technology, Santa Cruz, California, USA) (for antibody specificity, see [21]) and with CD31 monoclonal antibody (Serotec, Kidlington, UK), to test whether M3Rs were localized in salivary gland blood vessels. CD31 was used as a panendothelial marker. The secondary antibodies used were Cy3 conjugated goat-anti rabbit (Jackson Immuno Research, Baltimore, MD, USA) and Alexa Fluor Gold 488 conjugated goat anti-rat (Molecular Probes, Invitrogen, Paisley, UK). The sections were evaluated in a fluorescence microscope (Zeiss Axio Imager HBO 100; Carl Zeiss MicroImaging Inc., Jena, Germany).

### Immunohistochemistry

Six NOD mice and six BALB/c mice (Taconic; see Table 1) were anaesthetized as described above and were transcardiacally perfused with heparinized saline followed by fixative (4% paraformaldehyde with 0.2% picric acid in 0.1 M phosphate buffer, pH 7.4). The submandibular glands were excised and post-fixed for 2 hours. After cryoprotection with 30% sucrose, the glands were stored at -80°C until sectioning.

Alternate cryostat serial sections (30 μm) of the glands were processed for immunohistochemistry on precoated glass slides (SuperFrost Plus; Menzel-Glaser, Braunschweig, Germany). For staining of sympathetic and parasympathetic nerve fibres, alternate serial sections were incubated for 72 hours either with polyclonal rabbit anti-NPY antibody (1:4,000 dilution; Peninsula Laboratories Inc., San Carlos, California, USA) or with polyclonal rabbit anti-VIP antibody (1:5,000 dilution; Eurodiagnostica, Malmö, Sweden). The sections were rinsed in PBS and treated with 0.3% hydrogen peroxide in absolute methanol.

Sections for NPY labelling were incubated in 2.5% normal goat serum (Vector Ltd., Burlingam, CA, USA) for 1 hour, before NPY antibody (1:4,000) was incubated with 2.5% normal goat serum for 72 hours at 4°C. After several rinses in PBS, sections were incubated for 1 hour with biotinylated anti-rabbit immunoglobulin G (1:1,000; Vector). Following several PBS rinses, sections were incubated with ABC reagent (Vector) for 1 hour. Final visualization for NPY antibody was made with nickel-enhanced 0.025% 3,3'-diaminobenzidine tetrahydrochloride (Sigma-Aldrich, Inc., St Louis, MO, USA) with 0.1% H_2_O_2 _as the chromogen.

Sections incubated with anti-VIP antibody were left overnight before visualization with horseradish peroxidase-conjugated Envision^+® ^(Dako Cytomation, Carpinteria, CA, USA) with diaminobenzidine as the chromogen.

All sections were counterstained in Richardson's stain, and coverslipped with Assistent Histokitt (Assistant, Osterode, Germany).

### Negative controls

Controls of the specificity of the immunoreactions were routinely included by isotype control immunoglobulin incubation and by preabsorption of the primary antibody with its respective antigen.

### Evaluation of salivary gland inflammation

Salivary gland tissue sections from Taconic mice (*n *= 20) and sections of submandibular glands in Group 2 (*n *= 14) were evaluated to determine the degree of inflammation (Table 1). Sections (5 μm) were obtained using a cryostat (Leica Instruments, Nussloch, Germany) and were placed onto SuperFrost Plus glass slides (Menzel, Braunschweig, Germany). Haematoxylin and eosin staining was performed, and evaluation was performed in a representative section from each gland. Salivary gland sections were evaluated and morphometrically analysed using a Leica DMLB light microscope connected to a Color View III camera and Analysis software (Lucia v. 480; Laboratory Imaging, Hostivaø, Czech Republic) or AnalySIS^® ^software (Soft Imaging System, GmbH, Münster, Germany), to determine the focus score (that is, the number of foci comprising ≥ 50 mononuclear cells/mm^2 ^glandular tissue) and the ratio index (that is, the ratio of the area of inflammation to the total area of glandular tissue) [22,23].

### Statistical analyses

Results are presented as the mean ± standard error of the mean. Differences were tested between groups using the Students *t *test or the Mann–Whitney rank sum test. *P *< 0.05 was considered statistically significant.

## Results

### Blood flow responses to parasympathetic stimulation; effect of L-NAME (Group 1)

**Figure 1:**
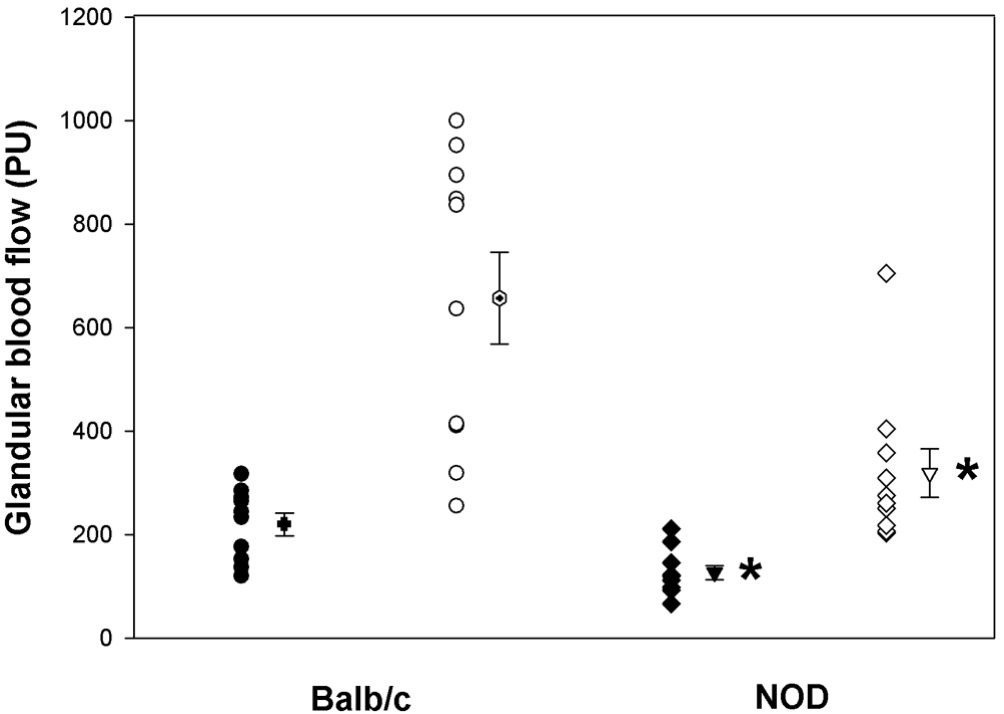
Individual glandular blood flow responses to parasympathetic nerve stimulation. Individual glandular blood flow measurements in perfusion units (PU) before (filled symbols) and after (open symbols) parasympathetic nerve stimulation of the BALB/c mice and nonobese diabetic (NOD) mice (Taconic Bomholtgård, Group 1). Also shown are mean ± standard error of the mean values for each group. *P < 0.005 when comparing the same experimental condition in BALB/c mice and NOD mice.

Baseline perfusion values were lower in NOD mice than BALB/c mice (128 ± 14 PU and 221 ± 22 PU, respectively; *P *= 0.002) (Figure 1), whereas the systemic blood pressure was higher in the NOD mice (69 ± 16 mmHg, *n *= 10) than in the BALB/c group (54 ± 16 mmHg, *n *= 10) although the difference was not significant (*P *= 0.054).

**Figure 2:**
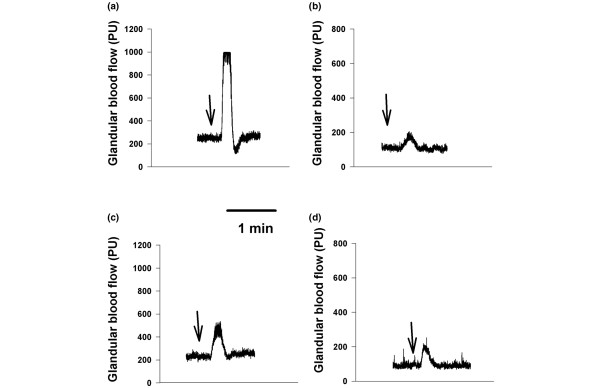
Responses in glandular blood flow after parasympathetic nerve stimulation and *N*_ω_-nitro-L-arginine-methyl ester infusion. **(a) **to **(d) **Original measurements in perfusion units (PU) (a, c) before and (b, d) after *N*_ω_-nitro-L-arginine-methyl ester L-NAME) infusion in (a, b) a BALB/c mouse and (c, d) a NOD mouse (Taconic Bomholtgård, Group 1). Start of electrical stimulation indicated by arrows (2 ms at 7 Hz, 8 V, 2 to 5 s).

**Figure 3:**
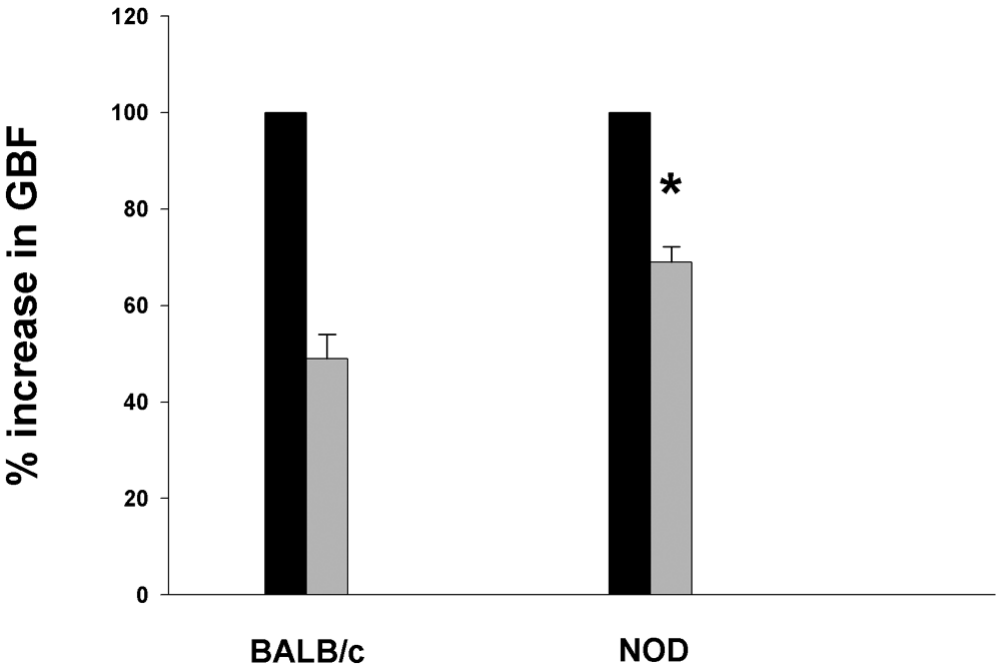
Effect of *N*_ω_-nitro-L-arginine-methyl ester infusion on glandular blood flow responses after parasympathetic nerve stimulation. Mean ± SEM glandular blood flow response (GBF) after parasympathetic nerve stimulation (%). Black columns, measurements before *N*_ω_-nitro-L-arginine-methyl ester (L -NAME) infusions; grey columns, measurements after L-NAME treatments. *P < 0.02 when comparing the difference between BALB/c mice and nonobese diabetic (NOD) mice.

When the parasympathetic nerve to the glands was stimulated, the maximal responses in glandular blood flow were significantly higher in BALB/c mice compared with NOD mice (Figure 1). In the BALB/c group the maximal responses averaged 656 ± 90 PU, compared with 319 ± 48 PU in the NOD group (Figure 1, *P *= 0.004) When L-NAME was administered a reduced blood flow response to parasympathetic stimulation was recorded in both groups as well (Figure 2). In BALB/c mice the mean reduction was -51 ± 5% PU, compared with -31 ± 3% PU in the NOD mice (*P *= 0.011, Figure 3).

### Blood flow responses to muscarinic receptor activation by pilocarpine (Group 2)

**Figure 4:**
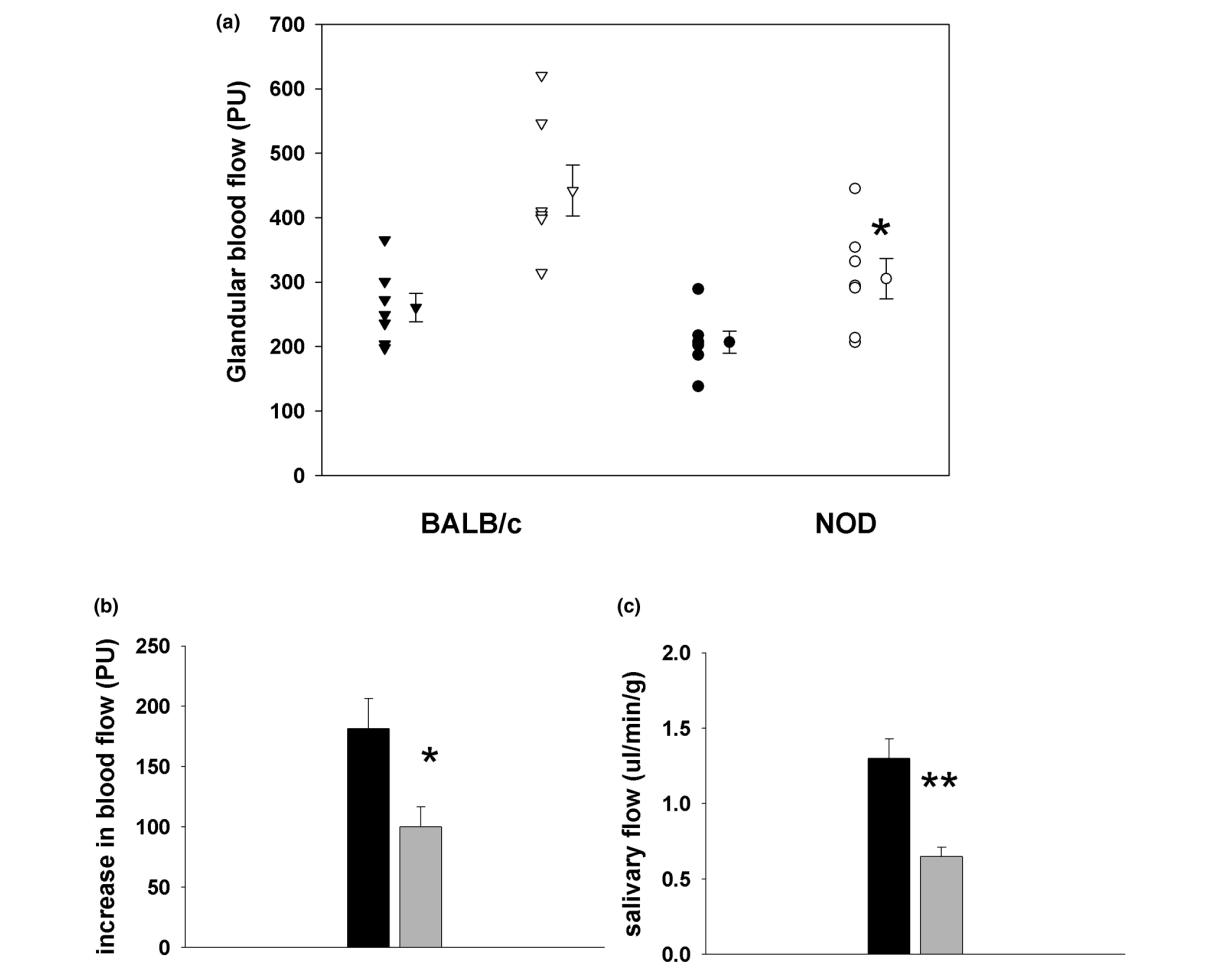
Effects of pilocarpine infusion on glandular blood flow and salivary flow rate. **(a) **Parallel individual glandular blood flow measurements, **(b) **mean increase in glandular blood and **(c) **salivary flow after pilocarpine infusion (0.1 mg/100 g body weight) in BALB/c and nonobese diabetic (NOD) mice (Jackson Laboratories, Group 2). (a) Glandular blood flow is measured in perfusion units (PU) before (filled symbols) and after (open symbols) pilocarpine infusion. (b, c) Also shown are mean ± standard error of the mean values for each group. BALB/c mice (*n *= 7, black columns) and NOD mice (*n *= 7, grey columns). *P < 0.05, **P < 0.005 when comparing the same experimental conditions in BALB/c mice and NOD mice.

Baseline perfusion values averaged 202 ± 17 PU and 261 ± 22 PU (*P *= 0.06, Figure 4a), and the systemic blood pressure was 67 ± 17 mmHg and 61 ± 15 mmHg (*P *= 0.52, *n *= 7) in NOD mice and BALB/c mice, respectively.

Immediately following pilocarpine infusion, an increase in blood flow was observed in both groups of animals. The increase averaged 181 ± 67 PU and 100 ± 44 PU, giving a maximal response of 442 ± 104 PU and 306 ± 83 PU in BALB/c mice and NOD mice, respectively (Figure 4a). The maximal blood flow responses (Figure 4a) as well as blood flow increases (Figure 4b) were significantly different between the groups (*P *= 0.02).

### Stimulated salivary secretion capacity

Salivary secretion after pilocarpine administration (0.05 μl/g BW) in the NOD mice from Taconic (*n *= 6) averaged 0.41 ± 0.15 μl/min/g, and in the BALB/c mice (*n *= 5) averaged 0.54 ± 0.18 μl/min/g. The difference was not statistically significant (*P *= 0.21).

In NOD mice from Jackson Laboratories (Group 2) a significant hyposecretion was found after pilocarpine administration (0.1 μl/g BW), compared with the BALB/c mice. The average secretion in BALB/c was 1.3 ± 0.33 μl/min/g, compared with 0.65 ± 0.16 μl/min/g in NOD mice (*P *= 0.001) (Figure 4c).

### Localization of M3R in submandibular glands

**Figure 5:**
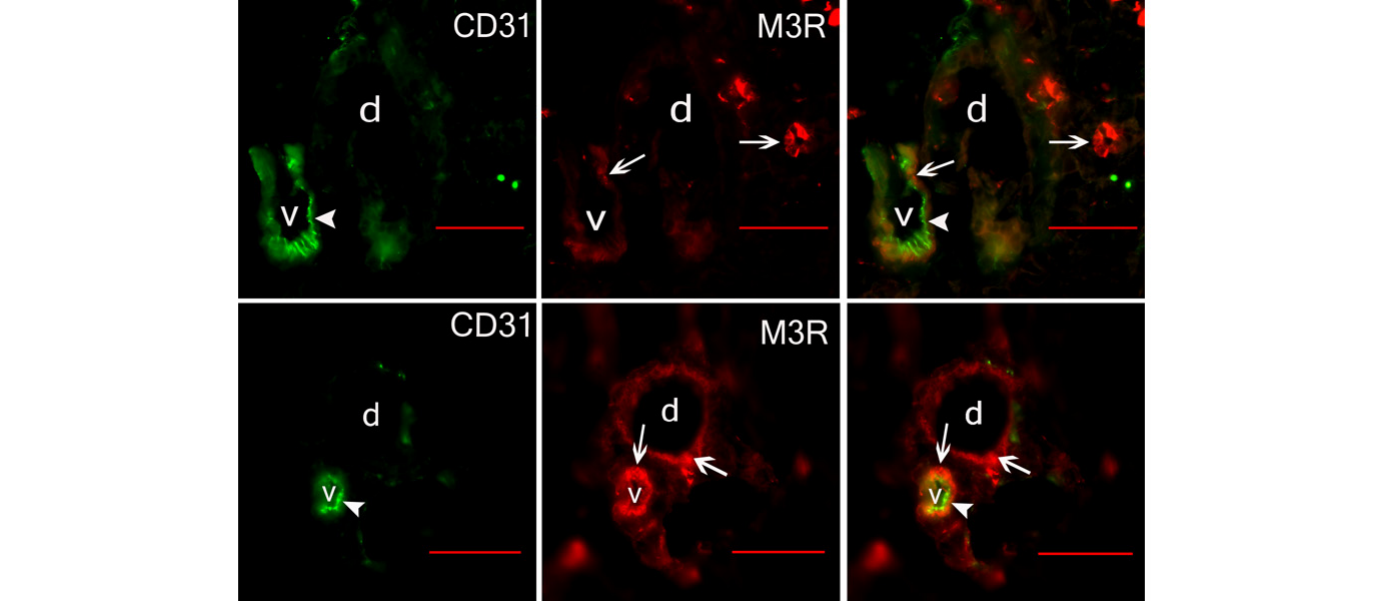
Localization of muscarinic 3 receptor in the walls of blood vessels in salivary glands. Fluorescent staining of CD31^+^/M3R^+ ^blood vessels in representative sections from submandibular glands of (top) a BALB/c mouse and (bottom) a nonobese diabetic (NOD) mouse (Jackson Laboratories, Group 2). Images showing M3R^+ ^staining (arrows) in the wall of CD31^+ ^blood vessels (arrowheads). M3R^+ ^acini cells (upper) and duct (lower) are also shown (arrows). Right images are merged. Scale bars = 50 μm. M3R, muscarinic 3 receptor; d, duct; v, vessel.

Double labelling of CD31 and M3R revealed M3R in the wall of blood vessels in submandibular glands of both NOD mice and BALB/c mice (Figure 5a,b). In addition, M3R staining was found in acinar and ductal epithelial cells (Figure 5a,b). CD31^+ ^blood vessels were usually seen adjacent to ducts in the submandibular glands of both strains (Figure 5a,b).

### Distribution of autonomic nerve fibres

**Figure 6:**
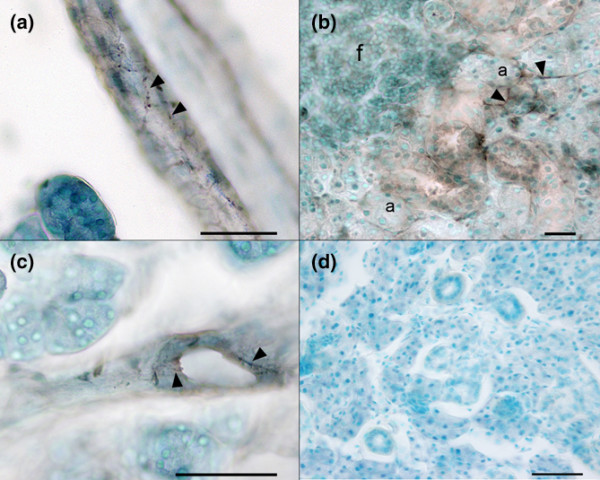
Distribution of vasoactive intestinal peptide-immunoreactive nerve fibres in submandibular glands. Light microscopic view of vasoactive intestinal peptide (VIP)-immunoreactive (IR) fibres in submandibular gland tissue from **(a)**, **(c)** a BALB/c mouse and **(b) **a nonobese diabetic (NOD) mice from Taconic Bomholtgård (Group 1). (a) Thin varicose fibres making a network in a blood vessel wall (arrowheads) in the central part of the gland. (b) VIP-IR fibres (arrowheads) in close proximity to acinar epithelial cells outside a focus of mononuclear cells. Note that the area of mononuclear cells lacks immunoreactivity to VIP. (c) A salivary duct supplied with thin VIP-IR fibres (arrows). **(d) **Control section from a NOD mouse after preabsorption of antibody with antigen. Scale bars = 50 μm. a, acinar cells; f, focus.

Immunohistochemical labelling of the submandibular gland tissue revealed thin VIP-positive nerve fibres surrounding blood vessels, and acinar and ductal epithelium, as illustrated in Figure 6a to 6c. The nerve fibres were seen throughout the glandular parenchyma, frequently surrounding the acinar epithelial cells. In areas surrounding the focal infiltrates, the staining pattern of the submandibular glands from NOD mice resembled that of BALB/c mice. VIP-positive nerve fibres were absent, however, from areas of focal infiltrates (Figure 6b).

**Figure 7:**
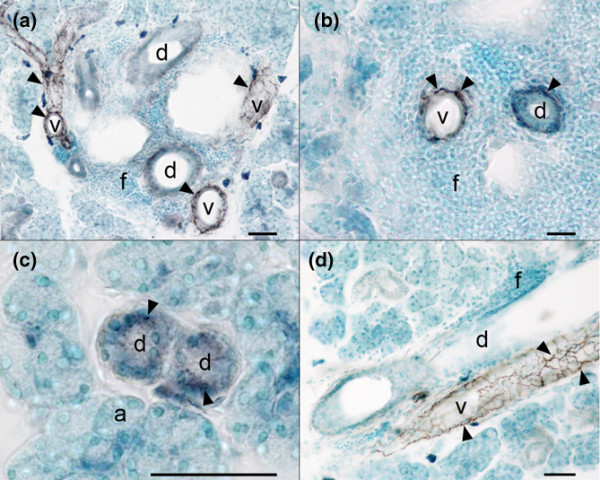
Immunolabelling of neuropeptide Y in blood vessels and ductal epithelium in the submandibular glands. Immunostaining with anti-neuropeptide Y (anti-NPY) antibody in sections from **(a)**, **(b)**, **(d)** nonobese diabetic (NOD) submandibular gland and **(c) **BALB/c submandibular gland (Taconic Bomholtgård mice, Group 1). Scale bars = 50 μm. (a) Numerous NPY-immunoreactive (IR) fibres (arrowheads) in walls of blood vessels in areas with focal mononuclear cell inflammation. (b) Immunoreactivity (arrowheads) in the wall of a blood vessel and a duct surrounded by a focus of infiltrating mononuclear cells. (c) Ductal cells showing intracellular NPY staining (arrowheads), whereas acini cells are without staining and lack innervation of NPY-IR fibres. (d) Typical localization of a vessel with a network of NPY-IR fibres in close proximity to a large duct (D) and a focus of infiltrating cells. a, acini; d, duct; f, focal mononuclear cell inflammation; v, vessel.

In contrast to VIP, NPY fibres were only detected around blood vessels, striated ducts and collecting ducts (Figure 7a,b,d). Around blood vessels, typically located close to collecting ducts, the NPY fibres formed plexuses (Figure 7d). Interestingly, immunolabelling for both NPY (Figure 7c) and VIP were observed in striated duct cells and may represent endogenous production of neuropeptides in these cells. Persisting blood vessels and ducts with innervating NPY fibres could still be detected in the inflammatory infiltrates (Figure 7b).

### Inflammation of the submandibular glands

Focal mononuclear cell infiltrates were observed in all salivary gland tissue samples from NOD mice, and ranged from 4 to 17 in Taconic mice and from 4 to 9 in Jackson mice. No such foci could be detected in the submandibular glands obtained from any BALB/c mice. The focus score in the NOD mice averaged 0.76 ± 0.09 and 0.63 ± 0.08, and the ratio index averaged 0.035 ± 0.005 and 0.018 ± 0.003 in animals from Taconic and Jackson Laboratories, respectively.

## Discussion

The NOD mouse strain manifesting focal mononuclear cell infiltrates and reduced stimulated saliva secretion is frequently used as a model for SS. The fluid component in saliva derives from the bloodstream, and blood flow in the salivary glands is tightly regulated by autonomic nerves. During parasympathetic nerve stimulation, vasodilatation and increased capillary blood pressure leads to increased filtration of fluid out of the glandular capillaries into the interstitial space before it is secreted as saliva by the glandular epithelium [24]. In patients with SS, the saliva secretion from the submandibular and sublingual glands is most severely affected [25]. Reduced blood flow responses to secretory stimulation has been reported in patients with SS, and may contribute to the reduced stimulated salivary gland output in this group of patients [26].

Functional blood flow studies have previously not, to our knowledge, been performed in NOD mice. In the present study, reduced responses to parasympathetic stimulation and to muscarinic receptor stimulation were recorded in submandibular glands in NOD mice compared with nondiseased controls (BALB/c mice). Our results indicate that NOD mice share the abnormal blood flow responses to parasympathetic stimulation described in SS patients. The altered blood flow responses in 17-week-old NOD mice (Group 2) observed after pilocarpine infusion were followed by a reduced salivary flow in this study. Whether changes in blood flow responses are aggravated and thereby cause or contribute to a more pronounced reduction in salivary flow later in the disease process is still elusive and needs further investigation. NOD mice from both suppliers developed focal infiltrates in the submandibular glands, whereas only the NOD mice from Jackson Laboratories revealed significantly lower salivary secretion rates as compared with BALB/c mice. Both colonies of NOD mice have been reported to develop hyposalivation as a consequence of salivary gland inflammation, but the Taconic animals develop hyposecretion [3] later in life than the Jackson Laboratories mice [27].

Inflammatory cytokines and chemokines can activate vascular cells and are suggested to be involved in atherogenesis [28]. In autoimmune diseases, vascular cells can actively contribute to the inflammatory cytokine-dependent network in the blood vessel wall. By interaction with invading cells, the activation may contribute to development of atherosclerosis and endothelial dysfunction [28]. The endothelial dysfunction may cause altered blood flow responses [29]. Whether such a dysfunction is a mechanistic factor for the impaired vascular response observed in NOD mice, however, is not known. A potential effect of diabetes in vascular disease development in the submandibular gland is avoided in the present study by using prediabetic mice.

Since part of the vasodilatation following parasympathetic nerve stimulation in salivary glands is mediated through NO release [30,31], we used a general inhibitor for NO synthesis (L-NAME) to elucidate whether endothelial dysfunction in submandibular glands is evident in NOD mice. Our results show that L-NAME treatment gave less reduction in blood flow responses to parasympathetic stimulation in NOD mice compared with BALB/c mice (Figures 2 and 3). This finding may indicate an endothelial dysfunction associated with a reduced NO synthesis activity in endothelial cells in NOD mice. L-NAME has also been demonstrated to have binding affinity to muscarinic receptors, and thereby acts as a muscarinic antagonist in addition to the ability to inhibit NO synthase [32]. It is therefore possible that part of the L-NAME effect observed in this study is due to a direct blocking of the muscarinic receptor. Consequently, a reduced blocking effect by L-NAME in NOD mice can at least in part be explained by changes in muscarinic receptor signalling. If this is the case, it provides support to the reduced effect in blood flow response observed after muscarinic receptor activation by pilocarpine in NOD mice.

Previous studies have shown alterations and progressive loss of NO synthesis activity in submandibular glands in NOD mice [16]. NO production by the vascular endothelium maintains an essential anti-inflammatory influence on the endothelial wall, including prevention of leukocyte–endothelial cell interactions probably mediated by downregulation of P-selectin [33]. Endothelial dysfunction with reduced NO production in the submandibular gland may contribute to the recruitment of inflammatory cells in SS. The endothelial dysfunction observed as reduced NO signalling in NOD mice may have contributed to the accumulation of inflammatory cells observed as focal mononuclear cell infiltrates.

It is also possible that NO has a basal tone on the vessels in the gland, and that a reduction in NO production can explain the relatively lower output values observed in basal blood flow in the submandibular gland in NOD mice compared with BALB/c mice.

In the current study we demonstrate for the first time the possible existence of M3R located in the blood vessel wall of the salivary gland in NOD mice. When the parasympathetic nerve innervating the gland is stimulated, acetylcholine and neuropeptides are released and bind to their corresponding receptors. In the salivary gland, muscarinic receptors are localized in blood vessels, myoepithelial cells, and acinar and ductal epithelial cells [13,34,35]. In a recent study, however, the suitability of muscarinic acetylcholine receptor antibodies for immunohistochemistry was evaluated on sections from receptor gene-deficient mice, and the results demonstrated uncertain specificity of muscarinic receptor subtype localization in tissue sections [36]. The use of preabsorbtion of the primary antibody with its respective antigen did not detect the nonspecificity, and the authors suggest that it might be due to stretches of amino acid sequences shared between two proteins. The phenomena often occur among members of a protein family or receptor isoforms. The results demonstrate that precautions must be taken when antibodies toward muscarinic receptors subtypes are utilized, and the detection of positive immunolabelling is not a final proof that this subtype of receptor is localized in the tissue.

Functional studies in rat parotid gland indicate that the vasodilatation in salivary glands may be mediated at least in part via muscarinic M3R [37]. The M3R density on acinar cells is reported to be altered in SS [11], and autoantibodies to the receptors can be detected in patients [38] as well as in NOD mice [12]. Contractile carbachol responses in smooth muscle cells were shown recently to be lower in NOD mice with circulating anti-M3R autoantibodies than in NOD mice lacking the same autoantibodies [39]. These results support the hypothesis that chronic stimulation of membrane-bound M3R can result in receptor desensitization leading to reduced responses. The similarly reduced blood flow responses after both parasympathetic nerve stimulation and pilocarpine infusion in this study may be explained by circulating autoantibodies, muscarinic receptor desensitization and/or reduced receptor density on the blood vessels walls. Another possible explanation is a defect in the intracellular signalling of target cells after muscarinic activation. Our finding indicates that the innervation of blood vessels by autonomic nerves is normal in NOD mice, supported by the notion that no visible differences were observed in the distribution of NPY and VIP immunoreactive nerve fibres around blood vessels in neither of the two NOD strains studied.

Reduced VIP concentrations in the submandibular gland of NOD mice have previously been reported together with reduced salivary secretion in response to VIP infusion [8], leading to the conclusion of a likely defective receptor mechanism leading to reduced neurotransmitter responsiveness. We did not, however, observe differences in VIP staining of blood vessels in the NOD mice compared with the BALB/c mice.

VIP-positive nerve fibres could not be detected in areas infiltrated by inflammatory cells, whereas in all other areas the innervation pattern resembled that observed in BALB/c mice (Figure 5b). This observation is in line with observations in SS patients where VIP fibres are depleted from central areas of focal lymphocytic infiltrates [9,40].

A tropic effect of VIP on salivary gland parenchyma has been postulated [40], and it is speculated whether the loss of innervation in areas of focal infiltrates is the forerunner of acinar epithelial cell atrophy in such areas. Our results in the present study support the speculation mentioned above, as we did not observe any VIP fibres in areas of focal infiltration.

VIP stimulates and potentiates salivary secretion in normal mice, but this ability is progressively lost in NOD mice [41]. Part of the VIP signalling effect is mediated by the NO/cGMP pathway, and VIP failed to increase cGMP in 14-week-old and 16-week-old NOD mice [41]. This finding leads to the conclusion that the reduced response to VIP is possibly due to a defect in the VIP-mediated signalling in the secreting cells.

Immunostaining of both VIP and NPY was observed in striated ductal epithelial cells (Figure 7c) in BALB/c and NOD submandibular glands. The role of this endogenous production of neuropeptides is unknown and requires further investigation. NPY has been observed close to the basal membrane in acinar epithelial cells in rat salivary glands [42], and seems to be of parasympathetic origin since they are significantly reduced after parasympathetic denervation [43]. In mouse submandibular glands, however, NPY-immunoreactive fibres are found only in the wall of blood vessels and ducts (Figures 7a,b,d). This finding supports the concept of two separate populations of sympathetic nerve fibres in salivary glands; one population involved in the control of blood flow, and the other associated with control of secretion. NPY was not found in parasympathetic fibres innervating acinar cells in submandibular salivary glands in NOD mice or BALB/c mice, and hence is seemingly uninvolved in the pathogenesis of acinar cell atrophy observed in SS.

## Conclusion

The NOD mouse is frequently used as an experimental model of SS, and the present study shows that the changes in distribution of parasympathetic nerves correspond with those observed in salivary glands in patients with SS. Furthermore, the present study provides new evidence of the pathogenesis of SS by demonstrating impaired blood flow responses in submandibular glands in NOD mice after parasympathetic nerve stimulation and muscarinic receptor activation. The latter was followed by a parallel reduction in salivary flow. The study also demonstrates dysfunctional endothelium with reduced NO signalling after parasympathetic stimulation in the NOD mice. The possible presence of M3R in the vasculature, reduced blood flow responses and salivary secretion to muscarinic receptor activation support the notion that M3R autoantibodies present in NOD mice might critically impair fluid transport required for salivation.

## Abbreviations

BW: body weight; L-NAME: *N*_ω_-nitro-L-arginine-methyl ester; M3R: muscarinic 3 receptor; NO: nitric oxide; NOD: nonobese diabetic; NPY: neuropeptide Y; PBS: phosphate-buffered saline; PU: perfusion units; SS: Sjögren's syndrome; VIP: vasoactive intestinal peptide.

## Competing interests

The authors declare that they have no competing interests.

## Authors' contributions

EB designed the study. EB and HH-I carried out the functional experiments. KN collected tissue and carried out the immunohistochemistry for neuropeptides, and EB carried out the immunofluorescent staining. EB carried out the data analyses. KN and MVJ carried out the focus score and ratio index analyses. EB, ND and MVJ wrote the manuscript. All authors read and approved the final manuscript.
